# Model-Agnostic Method for Thoracic Wall Segmentation in Fetal Ultrasound Videos

**DOI:** 10.3390/biom10121691

**Published:** 2020-12-17

**Authors:** Kanto Shozu, Masaaki Komatsu, Akira Sakai, Reina Komatsu, Ai Dozen, Hidenori Machino, Suguru Yasutomi, Tatsuya Arakaki, Ken Asada, Syuzo Kaneko, Ryu Matsuoka, Akitoshi Nakashima, Akihiko Sekizawa, Ryuji Hamamoto

**Affiliations:** 1 Division of Molecular Modification and Cancer Biology, National Cancer Center Research Institute, 5-1-1 Tsukiji, Chuo-ku, Tokyo 104-0045, Japan; kshozu@ncc.go.jp (K.S.); adozen@ncc.go.jp (A.D.); hidenori.machino@riken.jp (H.M.); ken.asada@riken.jp (K.A.); sykaneko@ncc.go.jp (S.K.); 2 Department of Obstetrics and Gynecology, University of Toyama, 2630 Sugitani, Toyama 930-0194, Japan; akinaka@med.u-toyama.ac.jp; 3 Cancer Translational Research Team, RIKEN Center for Advanced Intelligence Project, 1-4-1 Nihonbashi, Chuo-ku, Tokyo 103-0027, Japan; 4 Artificial Intelligence Laboratory, Fujitsu Laboratories Ltd., 4-1-1 Kamikodanaka, Nakahara-Ku, Kawasaki, Kanagawa 211-8588, Japan; akira.sakai@fujitsu.com (A.S.); yasutomi.suguru@fujitsu.com (S.Y.); 5 RIKEN AIP-Fujitsu Collaboration Center, RIKEN Center for Advanced Intelligence Project, 1-4-1 Nihonbashi, Chuo-ku, Tokyo 103-0027, Japan; rkomatsu@med.showa-u.ac.jp (R.K.); ryu@med.showa-u.ac.jp (R.M.); 6 Biomedical Science and Engineering Track, Graduate School of Medical and Dental Sciences, Tokyo Medical and Dental University, 1-5-45 Yushima, Bunkyo-Ku, Tokyo 113-8510, Japan; 7 Department of Obstetrics and Gynecology, Showa University School of Medicine, 1-5-8 Hatanodai, Shinagawa-Ku, Tokyo 142-8666, Japan; arakakit@med.showa-u.ac.jp (T.A.); sekizawa@med.showa-u.ac.jp (A.S.)

**Keywords:** deep learning, fetal ultrasound, prenatal diagnosis, thoracic wall segmentation, model-agnostic, ensemble learning

## Abstract

The application of segmentation methods to medical imaging has the potential to create novel diagnostic support models. With respect to fetal ultrasound, the thoracic wall is a key structure on the assessment of the chest region for examiners to recognize the relative orientation and size of structures inside the thorax, which are critical components in neonatal prognosis. In this study, to improve the segmentation performance of the thoracic wall in fetal ultrasound videos, we proposed a novel model-agnostic method using deep learning techniques: the Multi-Frame + Cylinder method (MFCY). The Multi-frame method (MF) uses time-series information of ultrasound videos, and the Cylinder method (CY) utilizes the shape of the thoracic wall. To evaluate the achieved improvement, we performed segmentation using five-fold cross-validation on 538 ultrasound frames in the four-chamber view (4CV) of 256 normal cases using U-net and DeepLabv3+. MFCY increased the mean values of the intersection over union (IoU) of thoracic wall segmentation from 0.448 to 0.493 for U-net and from 0.417 to 0.470 for DeepLabv3+. These results demonstrated that MFCY improved the segmentation performance of the thoracic wall in fetal ultrasound videos without altering the network structure. MFCY is expected to facilitate the development of diagnostic support models in fetal ultrasound by providing further accurate segmentation of the thoracic wall.

## 1. Introduction

In recent years, artificial intelligence (AI)-based models have been applied to problems in a wide range of fields, including medicine, with remarkable success [1,2]. In particular, image segmentation based on deep learning, which is an image processing method that labels each pixel of an object [3], has exhibited outstanding performance [4]. The application of segmentation methods to medical imaging has the potential to create novel support systems for clinical decisions [5,6]. For example, these features make image segmentation an effective strategy for building AI-based diagnostic support models with respect to fetal ultrasound [7,8].

Fetal ultrasound assessment of the chest region, especially with the four-chamber view (4CV), is essential because it contains the heart and lung, which are critical components in neonatal prognosis [9]. The thoracic wall, which surrounds the thoracic cavity and consists of multiple components, including the thoracic vertebra, ribs, sternum, and muscles [10], is a key structure to evaluate the 4CV frame (Supplementary Figure S1). By referring to the thoracic wall, examiners ensure that the obtained 4CV is in the appropriate cross-section [11], and then recognize the relative orientation and size of structures inside the thorax, which allows the detection of congenital abnormalities [12,13]. Thus, the thoracic wall segmentation can assist examiners in the identification of its area and orientation; it leads to develop feasible AI-based models for supporting the assessment of 4CV.

However, accurate segmentation of most anatomical structures in medical ultrasound is still challenging due to the low contrast between the target and background of the images [8]. In fact, our preliminary experiment of the segmentation of the heart, lung, and thoracic wall indicated that the segmentation performance of the existing convolutional neural network (CNN) models corresponding to the thoracic wall were suboptimal compared to the heart and lung (Appendix A Table A1).

In this study, to improve the segmentation performance of the thoracic wall, we proposed a new method called the Multi-Frame + Cylinder method (MFCY), which uses deep learning techniques. MFCY consists of the Multi-frame method (MF) using time-series information of ultrasound videos, and the Cylinder method (CY) utilizing the shape of the thoracic wall. MF and CY are also our original constituent methods. MFCY integrates only the prediction results obtained from arbitrary neural network models via ensemble learning. Consequently, MFCY is a model-agnostic method, which can be applied to any neural network models without modifying the network structure. Here, we demonstrated that MFCY improved the segmentation performance of the thoracic wall in fetal ultrasound videos in terms of several metrics.

### 1.1. Related Research

Two powerful and major CNNs have been used for image segmentation in recent years: U-net and DeepLabv3+. Ronneberger et al. developed U-net, based on a fully convolutional network, and achieved more accurate segmentation using smaller amounts of training data compared with the other methods [14]. U-net is particularly suitable for biomedical image segmentation. Several studies have reported superior segmentation performances using their models based on U-net [15]. Chen et al. developed DeepLabv3+ by combining pyramidal pooling modules with an encoder-decoder model and demonstrated its state-of-the-art performance on cityscape images [16]. In this study, the segmentation methods were based on U-net and DeepLabv3+ for comparing the performance of our proposed methods. We hereinafter refer to the use of U-net and Deeplabv3+ as the existing models.

Since image segmentation can automatically recognize the location and size of an object in pixels (Supplementary Figure S2), it plays a remarkable role in image analyses in various fields [17,18]. For instance, cityscapes segmentation is an essential technique for visual scene understanding and contributes to self-driving car technology [19]. In medical image analysis, organ and tumor segmentation works as a computer-aided diagnosis or detection in various modalities, such as computed tomography, magnetic resonance imaging, X-rays, histological images, and ultrasound [20]. Especially in fetal ultrasound, AI-based models using segmentation methods have a great potential to support clinicians. Several studies reported the segmentation of fetal structures, such as the head, heart, lung, whole thorax, placenta, amniotic fluid, and whole fetus, in fetal ultrasound images using different deep learning-based approaches [21,22,23,24,25]. Arnaout et al. performed segmentation of the whole thorax, heart, four cardiac chambers, and spine using U-net to calculate the cardiothoracic ratio, cardiac axis, and fractional area change [22]. Burgos-Artizzu et al. improved the performance of their software which estimates neonatal respiratory morbidity risk by utilizing fetal lung segmentation [23]. In this study, to the best of our knowledge, we attempted for the first time to focus on the thoracic wall segmentation in fetal ultrasound.

Several studies have employed time-series information of videos to improve the segmentation performance of fetal ultrasound. We previously employed neighboring frames in the module that calibrates segmentation results of ventricular septum calculated by CNN [26]. Yu et al. proposed a dynamic CNN fine-tuned with sequential frames for the segmentation of the fetal left ventricle [27]. However, MF is different from the aforementioned methods in that MF integrates the output of the thoracic wall segmentation from each neighboring frame in the manner of ensemble learning.

Ensemble learning is a powerful method that improves aggregate performance by combining the predictions obtained from multiple trained models [28]. Several researchers have proposed various ensemble learning-based methods for the segmentation of ultrasound images. Kusunose et al. calculated a probability score using a majority-voting ensemble of 10 CNNs to identify regional wall motion abnormalities in echocardiographic images [29]. We also utilized ensemble learning for both MF and CY to improve the segmentation performance of fetal thoracic wall in this study.

### 1.2. Our Contributions

The motivation for this study is to improve the segmentation performance of the cylindrical-shaped thoracic wall, which is frequently discontinuous in prediction labels. Please see Figures 2, 3b, and 4b in the Results section for concrete examples of the segmentation results. Accordingly, we utilized ensemble learning with multiple prediction labels associated with the thoracic wall segmentation. For this purpose, we applied two specific properties to prepare the prediction labels; one is the property that the ultrasound video is a series of images where neighboring images are very similar to each other, and the other is the property that the thoracic wall is always cylindrical. We expected that the prediction labels obtained with these properties could provide additional information via ensemble learning and improve the segmentation performance of the thoracic wall.

The contributions of our MF, CY, and MFCY are as follows:

- MF was designed to integrate the prediction labels, corresponding to each target image and its neighboring frames in the video, into a single prediction label. It takes advantage of the similarity between neighboring frames within the set of sequential time-series images that comprise a high frame-rate ultrasound video. As even slight cross-sectional variations between neighboring ultrasound images can affect the appearance of structures substantially. Thanks to this difference in appearance, it is possible to complement the thoracic wall area, which could not be recognized by a single segmentation if the area was recognized by the other segmentation. Collectively, the integration of multiple prediction labels of sequential images belonging to the same video complements the information with each other.
- CY integrates three prediction labels-thoracic wall, thoracic cavity, and whole thorax-for the thoracic wall segmentation. The three prediction labels are obtained from the three independent models trained by each annotation label. CY utilizes the prior knowledge that the thoracic wall is always cylindrical. Namely, the thoracic wall label could be obtained by hollowing out the thoracic cavity label from the whole thorax label. The output prediction label of the thoracic wall obtained by CY more firmly delineates the outer boundary by the whole thorax label and the inner boundary by the thoracic cavity label. Collectively, the three prediction labels from the three independent models can also complement the information with each other.
- MFCY, which is a combination of the two aforementioned methods, is a model-agnostic method. It functions by employing the predictions obtained from any CNN model. Therefore, it can be applied to any state-of-the-art deep learning technology, regardless of whether they are modified or not; it notes that there is no need to make modifications to the state-of-the-art deep learning technology to fit MFCY.

## 2. Materials and Methods

### 2.1. Outline of This Study

In this study, we developed MFCY to assist in the thoracic wall segmentation of 4CV. MF utilizes both the target images as well as their neighboring frames in the original videos and assimilates the corresponding predictions to achieve better aggregate performance. CY utilizes three predictions—the thoracic wall, the thoracic cavity, and the whole thorax. The thoracic wall, defined to be the exterior of the thorax, is the object of interest in this study, the thoracic cavity is defined to be the area enclosed by the thoracic wall, and the whole thorax is defined to be the union of the thoracic wall and the thoracic cavity. The integrated prediction corresponds to the area obtained by taking the union of the thoracic wall and the area obtained by removing the thoracic cavity from the whole thorax.

**Figure 1:**
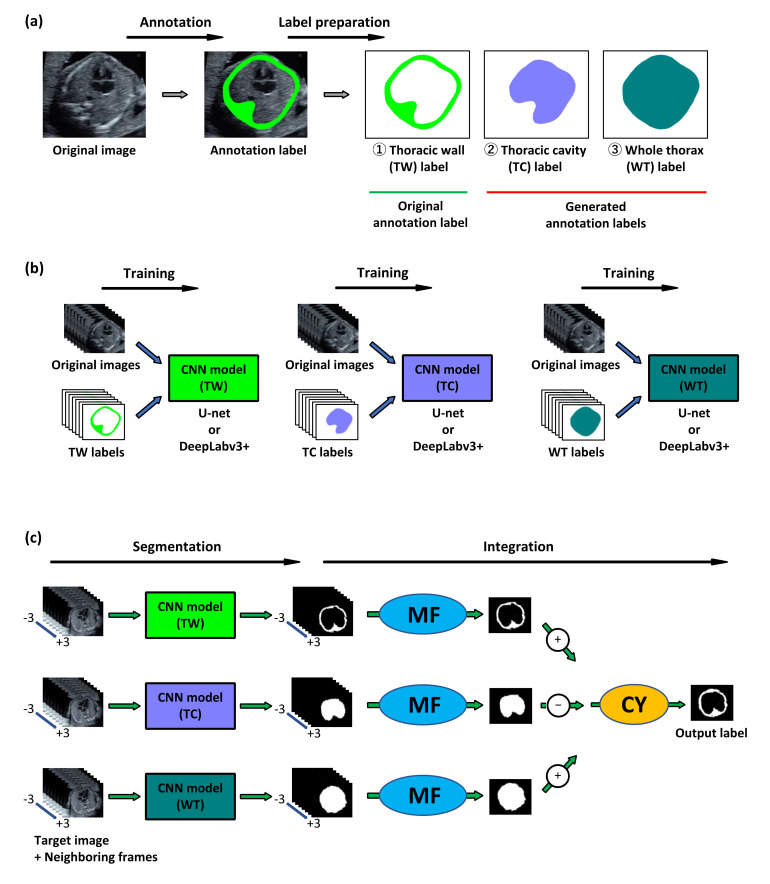
Outline of our proposed method, MFCY (Multi-Frame + Cylinder method). (**a**) Labels for supervised learning. Labels of the thoracic wall (TW) were annotated in the four-chamber-view (4CV) images extracted from fetal ultrasound videos. The thoracic cavity (TC) and the whole thorax (WT) labels were automatically generated based on the TW labels. These three labels were used for supervised learning. (**b**) Model training. Each convolutional neural network (CNN) model was trained using the original images and their respective labels. U-net and DeepLabv3+ were employed as CNNs. (**c**) Prediction. During the prediction phase, we extracted the target 4CV images and their neighboring frames from fetal ultrasound videos. These images were transmitted into the trained CNN models (TW, TC, and WT) to obtain the prediction labels corresponding to each image. The prediction labels corresponding to TW, TC, and WT were integrated into a single prediction label respectively via MF. Three independent labels were integrated using CY to obtain the prediction label which is the output of the thoracic wall segmentation using MFCY.

We examined the performance of MFCY using clinical fetal ultrasound videos. The outline of MFCY is shown in Figure 1. First, we prepared the labels of the thoracic wall, thoracic cavity, and whole thorax via thoracic wall annotation of the 4CV image extracted from fetal ultrasound screening video. The labels of the thoracic cavity and the whole thorax label were automatically generated based on the thoracic wall label according to their definitions. Second, segmentation models corresponding to the thoracic wall, thoracic cavity, and whole thorax were trained independently for CY. U-net and DeepLabv3+ were employed as CNNs. Finally, we performed segmentation using the three trained models and applied MF and CY to obtain the prediction label. In this phase, the target 4CV image and their neighboring frames extracted from fetal ultrasound videos were transmitted into the trained CNN models (thoracic wall, thoracic cavity, and whole thorax) to obtain the prediction labels corresponding to each image. These prediction labels were integrated using MF and CY (Supplementary Figure S3).

To validate the performance of MFCY, the tasks of the thoracic wall segmentation were also performed using the existing models (U-net and DeepLabv3+), MF, and CY, respectively (Supplementary Figure S4). We compared the performances of the existing models and our three methods (MF, CY, and MFCY) in terms of several metrics. In addition, we evaluated the effectiveness of MF and CY using individual cases. As supplementary experiments, we also performed the heart and lung segmentation using the existing models and MF, and compared their performance.

### 2.2. MF and CY

We describe MF and CY in detail. MF utilizes the neighboring frames of each target ultrasound image in an ultrasound video. Let $X_{t=0}$ be the target ultrasound image. Each element of $X_{t=0}$ is a real number between 0 and 1. Let $X_{t=t\in T}$ be its neighboring frames, where T denotes a set of neighboring instances of time. Furthermore, let a and k denote the frame interval and the factor of the frame number, respectively. Then, $T\left(a,k\right)=\left\{0,\;\pm a,\;\pm 2a,\;\ldots ,\;\pm ak\right\}$. The neighboring frames of $X_{t=0}$ are also denoted by real numbers between 0 and 1. The following element-wise step function is defined to be the threshold function:

(1) $$Y=H_{\phi }\left(X\right)\Leftrightarrow y_{ij}=\left\{\begin{array}{c} 0\;\left(x_{ij}<\phi \right)\\ 1\;\left(x_{ij}\geq \phi \right) \end{array}\right.,\;y_{ij}\in Y,\;x_{ij}\in X.$$

We consider that for a neural network, N, with a training parameter, θ, normalized network $\bar{N}\left(X;\theta \right)=H_{\phi =0.5}\left(N\left(X,\theta \right)\right)$. Each element of the output of N is assumed to be a real number between 0 and 1. Then, MF is given by:

(2) $$\mathrm{MF}\left(X_{t=t\in T};\bar{N},\;\theta ,\;\phi \right)=H_{\phi }\left(\underset{t\in T}{\sum }\bar{N}\left(X_{t};\theta \right)\right),$$

where ϕ denotes a threshold for MF and θ is obtained based on a set of $X_{t=0}$ and corresponding labels $Y_{t=0}$.

CY takes advantage of the topological shape of the subject of segmentation-a thick-walled cylinder. In this case, let X denote the ultrasound image. For a neural network, N, with a training parameter, θ, we use the following three trained parameters—$\theta_{TW}$, obtained using the thoracic wall label, $Y_{ET}$; $\theta_{TC}$, obtained using the thoracic cavity label, $Y_{TC}$; and $\theta_{WT}$, obtained using the whole thorax label, $Y_{WT}$. It is evident that $Y_{TW}=Y_{WT}-Y_{TC}$, and that $Y_{WT}$ and $Y_{TC}$ uniquely determine $Y_{TW}$. Element-wise subtraction is utilized in the aforementioned equation. Now, let us consider the learning parameters, $\Theta =\left\{\theta_{TW},\theta_{TC},\theta_{WT}\right\}$, and their threshold values. If $\Phi =\left\{\phi_{1},\phi_{2}\right\}$, then CY is given by:

(3) $$\mathrm{CY}\left(X;\bar{N},\;\Theta ,\;\Phi \right)=H_{\phi_{1}}\left(\bar{N}\left(X;\theta_{TW}\right)+H_{\phi_{2}}\left(\bar{N}\left(X;\theta_{WT}\right)-\bar{N}\left(X;\theta_{TC}\right)\right)\right),$$

where “+” denotes element-wise addition.

Finally, the combination of MF, CY, and MFCY is given by:

(4) $$\mathrm{MFCY}\left(X_{t=t\in T};\bar{N},\;\Theta ,\;\Phi \right)=H_{\phi_{1}}\left(\mathrm{MF}\left(X_{t=t\in T};\bar{N},\;\theta_{ET},\;\phi_{3}\right)+H_{\phi_{2}}\left(\mathrm{MF}\left(X_{t=t\in T};\bar{N},\;\theta_{WT},\;\phi_{3}\right)-\mathrm{MF}\left(X_{t=t\in T};\bar{N},\;\theta_{CT},\;\phi_{3}\right)\right)\right),$$

where $\Phi =\left\{\phi_{1},\;\phi_{2},\;\phi_{3}\right\}$ denotes the threshold values.

### 2.3. Network Architecture

U-net, which has a U-shaped structure, is constructed by adding a connection to the standard encoder-decoder structure to couple layers close to the input with those close to the output [14]. We adopted the improved version of U-net proposed in pix2pix [30]. In this variant, the encoder consisted of convolutional layers, and the decoder consisted of convolutional layers and a single up-sampling layer. The input and output sizes were 256 × 256 pixels, and the activation function was a rectifier unit. Each input element was a real number between −1 and 1, and each output was a real number between 0 and 1. Dice loss was adopted as the loss function for the segmentation. For further details, please consult Supplementary Figure S5.

DeepLab, which utilizes atrous convolution, is a high-performance segmentation method [31]. DeepLabv3+ is the latest version, which features a combination of encoder-decoder structures [16]. In this study, Resnet101, pretrained using ImageNet, was used as the backbone network for the encoder and cross-entropy is adopted as the loss function. The input and output sizes were 513 × 513 pixels. Each input element was a real number between −1 and 1, and each output was a real number between 0 and 1. For further details, please consult the reference [16]. 

### 2.4. Preparation of the Images and Labels

The study protocol was approved by the Institutional Review Boards of RIKEN, Fujitsu Ltd., Showa University, and the National Cancer Center (approval ID: Wako1 29–4). All methods were carried out in accordance with the declaration of Helsinki and the Ethical Guidelines for Medical and Health Research Involving Human Subjects. The participants of this study were singleton pregnant women with normal fetuses who had undergone fetal ultrasound screening during their second trimester at four Showa University Hospitals (Tokyo and Yokohama, Japan) between April, 2018 and May, 2019. The pediatricians confirmed that their normal fetuses are free of congenital diseases based on clinical findings at least one month after birth. Fetal ultrasound examination was performed by obstetricians with varying levels of expertise using Voluson® E8 or E10 (GE Healthcare, Chicago, IL, USA) equipped with a transabdominal 2–6 MHz convex transducer and the settings corresponding to the fetal heart. Each fetal ultrasound video consisted of a sequence of cross-sections relative to the fetus, sweeping from the level of the stomach, past the 4CV, to the vascular arches. All frames of these videos were magnified until the chest filled at least one half to two-thirds of the screen. Following this protocol, we collected one or more videos from each case. Based on the obtained videos, we extracted the 4CV images with the quality to recognize the information needed to evaluate the 4CV. Following the above process, the dataset for fetal thoracic wall segmentation comprised 538 4CV images in 280 videos of 256 cases. The median number of gestational age for the 256 cases was 20 weeks (range: 18–28 weeks).

The thoracic wall labels were manually annotated in these images by an obstetrician. The thoracic wall was defined to be the thick-walled cylinder between the two following boundaries—the outer boundary, which included the ribs and spinal column but did not include any skin or outer muscles, and the inner boundary, which coincided with the pleura. This definition was based on the Guidelines for fetal echocardiography published by the Fetal Echocardiography Guidelines Committee, Japanese Society of Fetal Cardiology and Japan Association of Pediatric Cardiology and Cardiac Surgery [13].

CY requires three different models trained by three different annotation labels of the thoracic cavity, thoracic wall, and whole thorax, respectively. We need two additional labels (thoracic cavity and whole thorax) for CY, but we can automatically generate these two labels from the thoracic wall labels. This means additional annotation cost for CY is free. In addition, we extracted the neighboring frames of each target image following the configurations used in MF. All images and labels to be used as input data were resized to 256 × 256 pixels in the case of U-net and 513 × 513 pixels in the case of DeepLabv3+.

### 2.5. Evaluation Metrics and Cross-Validation

We utilized four metrics—intersection over union (IoU), Dice coefficient (Dice), Precision, and Recall—to evaluate the performance of the segmentation models [32]. If true positives are denoted by TP, false positives by FP, false negatives by FN, and false positives by FP, then IoU, Dice, Precision, and Recall can be defined as follows:

(5) $$\mathrm{IoU}=\frac{\mathrm{TP}}{\mathrm{TP}+\mathrm{FP}+\mathrm{FN}}$$

(6) $$\mathrm{Dice}=\frac{2\mathrm{TP}}{2\mathrm{TP}+\mathrm{FP}+\mathrm{FN}}$$

(7) $$\mathrm{Precision}=\frac{\mathrm{TP}}{\mathrm{TP}+\mathrm{FP}}$$

(8) $$\mathrm{Recall}=\frac{\mathrm{TP}}{\mathrm{TP}+\mathrm{FN}}.$$

IoU, also known as the Jaccard index, and Dice are the most commonly used metrics used to evaluate segmentation methods. Precision and recall, which are also commonly used, were included to assess the details of the predictions. All four aforementioned metrics take values between 0 and 1, with values closer to 1 corresponding to better predictions.

We utilized five-fold cross-validation to generalize the performances of the existing models and our three methods: MF, CY, and MFCY. To this end, the dataset was uniformly divided into five independent subsets such that they were disjointed and almost equal in cardinality. Five-fold cross-validation functions by repeating folds, each of which assigns one subset as the test dataset and sequentially assigns the remaining four subsets as the training datasets. We calculated the mean IoU (mIoU), Dice (mDice), Precision (mPrecision), and Recall (mRecall) for each test dataset. Finally, we calculated the five-fold mean and the population standard deviation of mIoU, mDice, mPrecision, and mRecall.

We also calculated the IoU of individual test images to evaluate the performance of MF or CY in detail. Let IoUU-net, IoUDeepLabv3+, IoUMF, and IoUCY, denote IoU of the thoracic wall segmentation by U-net, DeepLabv3+, MF, and CY, respectively. We evaluated the differences for the IoU values of them (ΔIoU = IoUMF − IoUU-net or IoUDeepLabv3+, or ΔIoU = IoUCY − IoUU-net or IoUDeepLabv3+). The ΔIoU were calculated in all test images in all five-fold. CNN models were based on U-net and DeepLabv3+.

### 2.6. Configurations Used in Experiments

During the segmentation process, data augmentation or fine-tuning were not conducted on U-net and DeepLabv3+ to ensure equal operating conditions for all candidate methods. In the case of U-net, the batch size was 12 and the epoch size was 40, whereas 30,000 iterations and a batch size of 8 were adopted in the case of DeepLabv3+. We employed an Adam optimizer and the learning rate was 0.001 in U-net. The stochastic gradient descent with Nesterov momentum of 0.9, an initial learning rate of 0.007, and a decay rate of 0.9 every 2000 iterations were adopted in DeepLabv3+. In the case of CY, we set $T\;\left(a,\;k\right)=T\;\left(3,3\right)$. We also set ϕ=0.5 in Equation (1), ϕ=3/7 in Equation (2), $\Phi \left\{\phi_{1},\;\phi_{2}\right\}=\left\{0.5,\;0.5\right\}$ in Equation (3), and $\Phi \left\{\phi_{1},\;\phi_{2},\;\phi_{3}\right\}=\left\{0.5,\;0.5,\;3/7\right\}$ in Equation (4). We utilized Keras ver2.2 (TensorFlow ver1.9 backend) for U-net, TensorFlow ver1.10 for DeepLabv3+. All experiments were performed on a computer cluster with CentOS Linux release 7.2, CUDA Version: 10. The hardware of each node was Intel(R) Xeon(R) CPU E5-2690 v4 at 2.60 GHz, GeForce GTX 1080 Ti. The source code of the method proposed in this study is available on GitHub at https://github.com/rafcc/2020-circle-segmentation.

## 3. Results

### 3.1. Comparison between the Performances of the Existing Models and Our Methods

**Figure 2:**
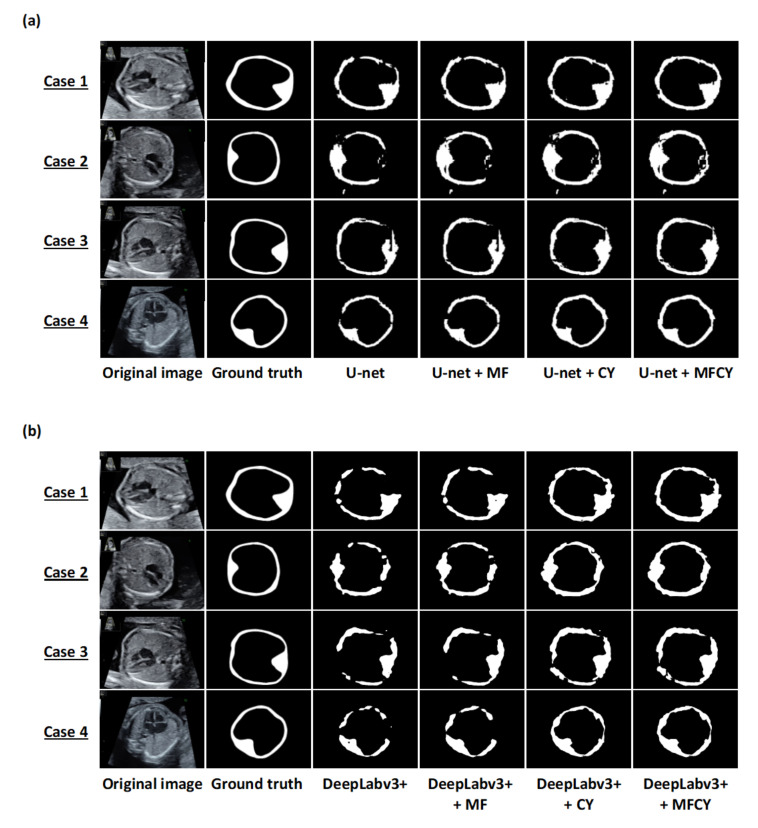
Representative examples of the thoracic wall segmentation by the existing models and our three methods on test datasets. Each row shows a particular case. The columns correspond to the original images, ground truths, and predictions by U-net, DeepLabv3+, MF, CY, and MFCY. The white areas represent the labels of the thoracic wall; (**a**) corresponds to U-net and (**b**) corresponds to DeepLabv3+.

We compared the thoracic wall predictions obtained via U-net, DeepLabv3+, MF, CY, and MFCY in terms of mIoU, mDice, mPrecision, and mRecall (Table 1). The predictions obtained via each model, along with the corresponding ground truths and prediction labels, are shown in Figure 2. MFCY performed better than the existing models in terms of mIoU—with scores of 0.448 (U-net) vs. 0.493 (U-net + MFCY), and 0.417 (DeepLabv3+) vs. 0.470 (DeepLabv3+ + MFCY). mRecall of the MFCY was also observed to be higher than that of the existing models—with scores of 0.568 (U-net) vs. 0.738 (U-net + MFCY), and 0.525 (DeepLabv3+) vs. 0.729 (DeepLabv3+ + MFCY). However, mPrecision of MFCY was observed to be lower than that of the existing models—with scores of 0.679 (U-net) vs. 0.596 (U-net + MFCY), and 0.675 (DeepLabv3+) vs. 0.566 (DeepLabv3+ + MFCY). The results corresponding to each fold are shown in Supplementary Figure S6. In addition, the predictions of the thoracic cavity and whole thorax obtained using MF are included in Supplementary Table S1. We also evaluated the performances of MF corresponding to the segmentation of the heart and lung (Supplementary Table S2). Our results showed that MF is effective to provide further accurate segmentation for not only the thoracic wall, thoracic cavity, and whole thorax, but also the heart and lung.

### 3.2. Analysis of Individual Effectiveness of MF and CY

**Figure 3:**
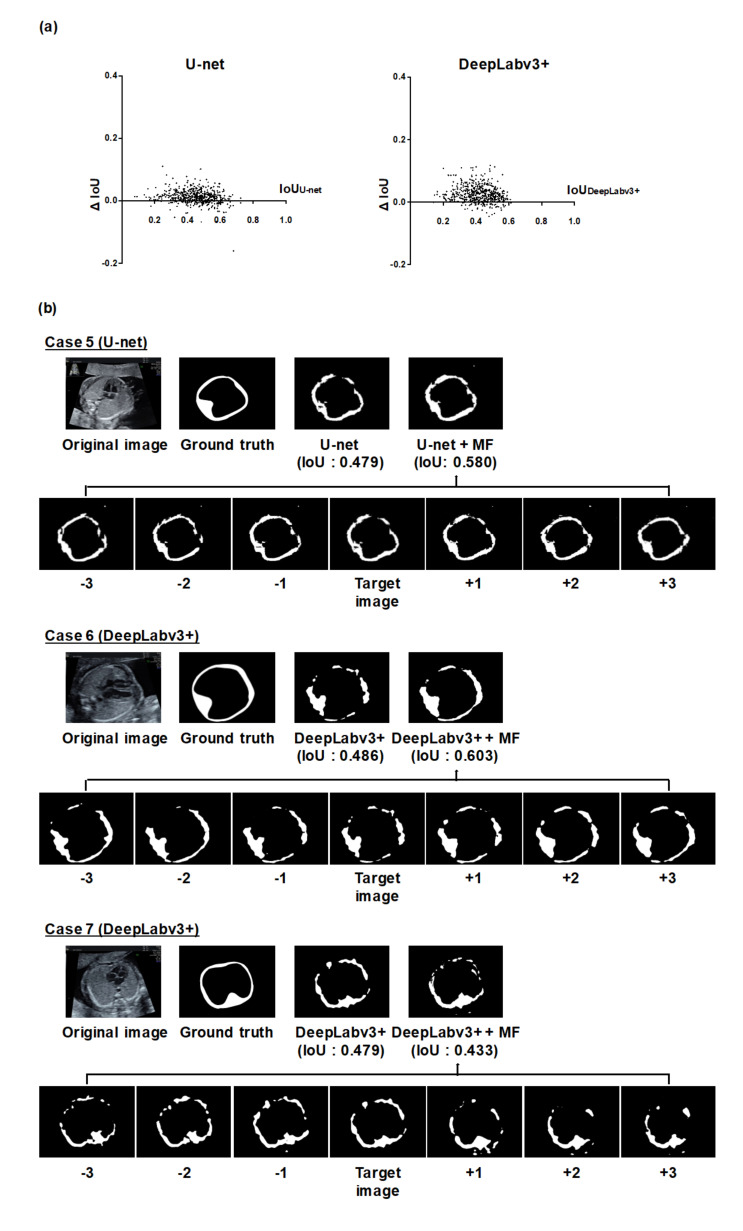
Analysis of the thoracic wall segmentation in the IoU metric using MF. (**a**) The differences of the IoU values in all individual images using U-net or DeepLabv3+ and MF. We calculated the IoU of U-net or DeepLabv3+ (IoUU-net or IoUDeepLabv3+), the IoU of MF (IoUMF), and the difference for the IoU values between them (ΔIoU = IoUMF − IoUU-net or IoUDeepLabv3+). CNN models were based on U-net and DeepLabv3+. The x-axis represents the IoUU-net or IoUDeepLabv3+, and the y-axis represents ΔIoU. (**b**) Representative examples of the thoracic wall segmentation using MF. CNN models were based on U-net (case 5) and DeepLabv3+ (case 6, 7). The upper row of each case presents the prediction labels obtained from U-net or DeepLabv3+ alone and MF. The lower row shows the prediction labels of the neighboring frames integrated by MF. In case 5 and 6, the segmentation performance was improved by MF. On the other hand, in case 7, the segmentation performance worsened by MF.

**Figure 4:**
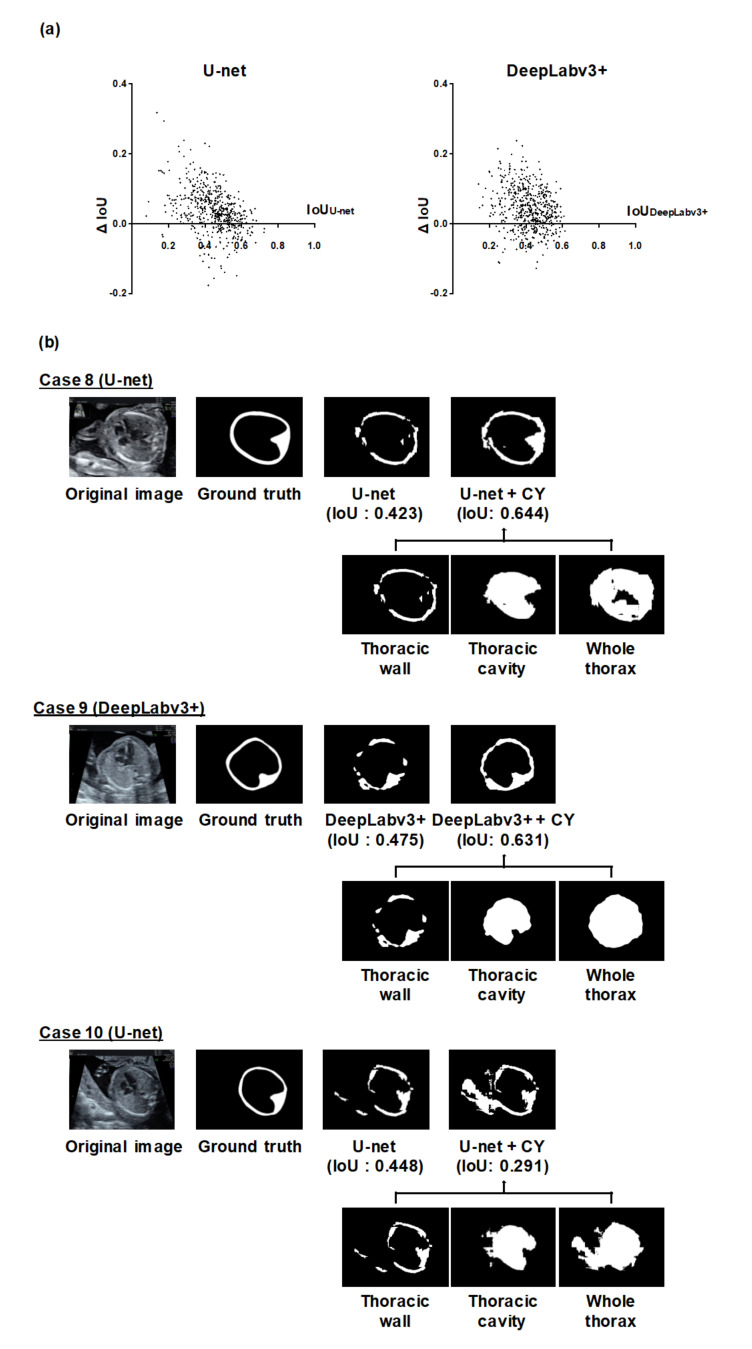
Analysis of the thoracic wall segmentation in the IoU metric using CY. (**a**) The differences of the IoU values in all individual images using U-net or DeepLabv3+ and CY. We calculated the IoU of U-net or DeepLabv3+ (IoUU-net or IoUDeepLabv3+), the IoU of CY (IoUCY), and the difference for the IoU values between them (ΔIoU = IoUCY − IoUU-net or IoUDeepLabv3+). CNN models were based on U-net and DeepLabv3+. The x-axis represents the IoUU-net or IoUDeepLabv3+, and the y-axis represents ΔIoU. (**b**) Representative examples of the thoracic wall segmentation using CY. CNN models were based on U-net (case 8, 10) and DeepLabv3+ (case 9). The upper row of each case presents the prediction labels obtained from the U-net or DeepLabv3+ alone and CY. The lower row shows the prediction labels of the neighboring frames integrated by CY. In case 8 and 9, the segmentation performance was improved by CY. On the other hand, in case 10, the segmentation performance worsened by CY.

Next, we analyzed the improvement of the thoracic wall segmentation using MF or CY to assess their individual contributions (Figure 3 and Figure 4 and Supplementary Figures S7 and S8). The improvement of segmentation results depended on each image (Figure 3a and Figure 4a). Case 5 and 6 in Figure 3b and case 8 and 9 in Figure 4b show the typical improved examples. In these cases, both MF and CY compensated for the failing segmentation areas of the existing models. Case 7 in Figure 3b and case 10 in Figure 4b show the typical worsened examples. In case 7, the integrated prediction became worse because the prediction result of the neighboring frames for MF appeared to be poor. In case 10, the incorrect segmentation result caused by the objects outside of the thorax worsen the integrated prediction by CY.

## 4. Discussion

Thoracic wall is a key structure on fetal ultrasound examinations to detect and estimate congenital abnormalities. Firstly, examiners validate that their scanning view is in the appropriate cross-section by obtaining the symmetrical thoracic wall, which has a full length of a rib in each side [11]. Secondly, the variation of the thoracic wall shape indicates some congenital abnormalities. In particular, a small thoracic circumference is present in cases of skeletal dysplasia with narrow thorax [33], and the thoracic wall itself is deformed in cases of pectus excavatum [34]. Thirdly, certain indicators for the detection and prognosis of congenital abnormalities are calculated by measurement of the thoracic wall. For example, the cardiothoracic area ratio, the ratio of the area of the heart and the thorax on 4CV, is a representative indicator of congenital heart diseases (CHDs) and fetal heart failure [35,36]. The lung to thorax transverse area ratio, the ratio of the area of the contralateral lung to that of the thorax on 4CV, is also a prognostic indicator of congenital diaphragmatic hernia [37,38]. Finally, cardiac axis and position, determined by the orientation and location of the heart relative to the thoracic wall, can trigger the detection of fetal abnormalities [12,13]. Thus, AI-based models adopting the thoracic wall segmentation have the potential to support the assessment of 4CV and the diagnosis of congenital abnormalities in fetal ultrasound. In this respect, MFCY can contribute to the progress of AI-based diagnostic support models in fetal ultrasound videos by providing accurate segmentation of the thoracic wall.

In this study, we proposed MFCY, a novel segmentation method in fetal ultrasound videos by combining two model-agnostic methods. MFCY exhibited superior segmentation performance of the fetal thoracic wall to the existing models. The mIoU and mDice of the thoracic wall segmentation using MFCY were observed to be higher than those using the existing models. MFCY works by applying the predictions of the existing neural networks with no modification and integrating the predictions of the target images and their neighboring frames in each video. Remarkably, we improved segmentation performance without making any modifications to the network or requiring any effort for greater annotation, even though the dataset was limited. Therefore, MFCY can be applied to other network architectures. Broad applicability to other segmentation methods is an advantage of any model-agnostic method.

Next, we evaluated the detailed contributions of MF or CY to the improvement of the thoracic wall segmentation based on the current results. We confirmed that the performance of thoracic wall segmentation is better than the existing models even when using MF or CY individually. The mPrecisions of MF, CY, and MFCY were observed to be lower than that of the existing models, whereas their mRecalls were observed to be higher. This can be attributed to the broad area covered by MF due to its utilization of a series of slightly shifted images. Additionally, the covered area was likely to be more extensive than the corresponding ground truth label, due to the mathematical operations (Equation (3)) required to obtain the integrated prediction in CY. Both MF and CY often improved segmentation results by compensating for failing areas and connecting the segmentation area closer to the cylindrical shape. This can be attributed to the compensation arising from the ensemble learning of the independent predictions of MF and CY. In MF, the utilization of neighboring frames corresponding to each target image improves the segmentation performance by compensating for failing areas that do not necessarily appear in every neighboring frame (e.g., case 5 and 6 in Figure 3b). Since the segmentation performance is affected by the temporal and spatial variation in the thoracic wall appearance, the failing area in the target image can be compensated with the information of the neighboring frames that identify the pixels of the failing area. In CY, the integration of output from three different models induces better performance than the implementation of any single model. Based on the prior knowledge that the thoracic wall has a cylindrical structure, multiple labels (thoracic cavity label and whole thorax label) can be generated from a single label (thoracic wall label). The other way around, prediction labels of the thoracic cavity and the whole thorax can determine a prediction label of the thoracic wall (e.g., case 8 and 9 in Figure 4b). Although the size of the dataset was limited in this study, it is considered that our methods are effective from these qualitative analyses

From another perspective, we considered the reasons for the difficulty of the thoracic wall segmentation in fetal ultrasound. In the cases of poor thoracic wall segmentation of U-net and DeepLabv3+, prediction labels of the thoracic wall were often interrupted (Figure 2, Figure 3b and Figure 4b). One possible reason was the temporal and spatial variation in the thoracic wall appearance, even within the same fetal ultrasound video. The appearance of the thoracic wall varies in fetal ultrasounds, because it is a mixture of structures of diverse brightness, and artifacts frequently appear in the thoracic wall, which are caused by the wall itself or the surrounding structures. In addition, the thoracic wall is not a simply connected topological space; thus, the necessity to identify its exterior region and inner hole further complicates the segmentation procedure. For these reasons, it is probable that we obtained suboptimal prediction results from the simple use of U-net or DeepLabv3+.

### Limitations

This study suffers from certain limitations. First, we verified the performance of our proposed MFCY only in conjunction with the representative models, U-net and DeepLabv3+. Since MFCY is a model-agnostic method, it is formally applicable to all deep learning algorithms. However, it is unclear how much segmentation performance can be improved by adapting MFCY to other deep learning algorithms. Second, the input data were acquired from only two types of ultrasonography machines operated by a single company, which does not ensure the robustness of the equipment. Thus, further experimentation is required to determine whether MFCY can be adapted to datasets acquired from various devices. Third, the dataset in this study included ultrasound videos of only normal fetuses, which means that we have not examined the effectiveness of MFCY in the cases of fetal abnormalities such as CHDs. The establishment of a general AI-based model also requires the inclusion of both normal and abnormal cases. Fourth, we focused on the thoracic wall segmentation in this study and cannot validate the effectiveness of MFCY for other structures using our dataset. To show the importance and expand the application of MFCY, we should test the segmentations of other organs with a cylindrical shape such as the fetal skull. Finally, the validation in clinical settings was out of scope in this study; the maximum mIoU (U-net + MFCY) was 0.493, which might be still relatively low for a clinical utility. Increasing the number of training data is expected to provide further accurate segmentation. Therefore, the implementation and validation of MFCY in a clinical scenario is a topic for future work.

## 5. Conclusions

This study demonstrated that our model-agnostic method, MFCY, improved the segmentation performance of the thoracic wall in fetal ultrasound videos. MFCY is based on ensemble learning of the independent predictions of the two methods; it complements time-series information of ultrasound videos and the shape information of the thoracic wall each other. Accurate segmentation of the fetal thoracic wall is essential to construct AI-based models for supporting the assessment of 4CV. MFCY is expected to lead to the development of automatic diagnostic support technologies in fetal ultrasound.

## Figures and Tables

**Table 1:** Thoracic wall segmentation performance of each set of combinations of MF and CY with the two CNNs.

CNN	MF	CY	mIoU		mDice		mPrecision		mRecall	
			Mean	SD	Mean	SD	Mean	SD	Mean	SD
U-net			0.448	0.017	0.610	0.016	0.679	0.033	0.568	0.032
	✓		0.461	0.017	0.623	0.016	0.659	0.035	0.606	0.032
		✓	0.486	0.006	0.647	0.005	0.599	0.014	0.718	0.025
	✓	✓	0.493	0.006	0.654	0.005	0.596	0.016	0.738	0.023
DeepLabv3+			0.417	0.007	0.582	0.007	0.675	0.009	0.525	0.013
	✓		0.443	0.006	0.608	0.006	0.663	0.009	0.575	0.014
		✓	0.462	0.005	0.626	0.005	0.567	0.004	0.709	0.012
	✓	✓	0.470	0.004	0.633	0.004	0.566	0.007	0.729	0.010

mIoU: the mean value of intersection over union; mDice: the mean value of Dice; mPrecision: the mean value of Precision; mRecall: the mean value of Recall; SD: standard deviation.

## Supplementary Materials

The following are available online at https://www.mdpi.com/2218-273X/10/12/1691/s1, Figure S1: Examples of clinical procedures that need to consider the thoracic wall when assessing the four-chamber view (4CV) in fetal ultrasound, Figure S2: The typical image processing methods using deep learning techniques, Figure S3: Schematic diagrams of procedures for MF and CY, Figure S4: Experimental diagrams for evaluation of MF, CY, and MFCY, Figure S5: Architecture of U-net, Figure S6: Thoracic wall segmentation performance with five-fold cross-validation, Figure S7: The differences for the IoU values of the thoracic wall segmentation in individual test images in each five-fold using MF, Figure S8: The differences for the IoU values of the thoracic wall segmentation in individual test images in each five-fold using CY, Table S1: The performances of MF corresponding to the segmentation of the thoracic wall, thoracic cavity, and whole thorax, Table S2: The performances of MF corresponding to the segmentation of the heart and lung.

## Appendix A

The segmentation of the heart, lung, and thoracic wall by the existing model was evaluated. The dataset, comprising 538 4CV images of 256 normal cases, was identical to the one described in the main text. The heart, lung, and thoracic wall labels were manually annotated in these images by an obstetrician. U-net and DeepLabv3+ were used as the existing models. U-net was used with a batch size of 12 and the epoch size of 40, whereas 30,000 iterations and a batch size of 8 were adopted in DeepLabv3+. Architecture of U-net was identical to that described in the main text (Supplementary Figure S5). For more details on DeepLabv3+, please refer to [16]. Segmentation was performed with five-fold cross-validation. Table A1 lists the results of the experiment. The mIoU values of segmentation in the cases of the heart, lung, and thoracic wall were 0.686, 0.712, and 0.448, respectively, in U-net, and 0.734, 0.684, and 0.417, respectively, in DeepLabv3+. The segmentation performance of the existing CNN models corresponding to the thoracic wall were suboptimal compared to the heart and lung.

**Table A1:** The performance of the segmentation of the heart, lung, and thoracic wall using U-net and DeepLabv3+.

CNN	Structure	mIoU		mDice		mPrecision		mRecall	
		Mean	SD	Mean	SD	Mean	SD	Mean	SD
U-net	Heart	0.686	0.042	0.798	0.036	0.849	0.028	0.784	0.069
	Lung	0.712	0.015	0.828	0.011	0.832	0.020	0.832	0.014
	Thoracic wall	0.448	0.017	0.610	0.016	0.679	0.033	0.568	0.032
DeepLabv3+	Heart	0.734	0.012	0.842	0.009	0.864	0.008	0.833	0.008
	Lung	0.684	0.010	0.808	0.008	0.833	0.008	0.793	0.014
	Thoracic wall	0.417	0.007	0.582	0.007	0.675	0.009	0.525	0.013

SD = standard deviation.
