# Supplementary material for: Model-Agnostic Method for Thoracic Wall Segmentation in Fetal Ultrasound Videos
Source: Biomolecules. 2020 Dec 17;10(12):1691. doi: 10.3390/biom10121691 (PMC7766150; doi:10.3390/biom10121691)
Supplement: Supplementary file 1 [file biomolecules-10-01691-s001.pdf]

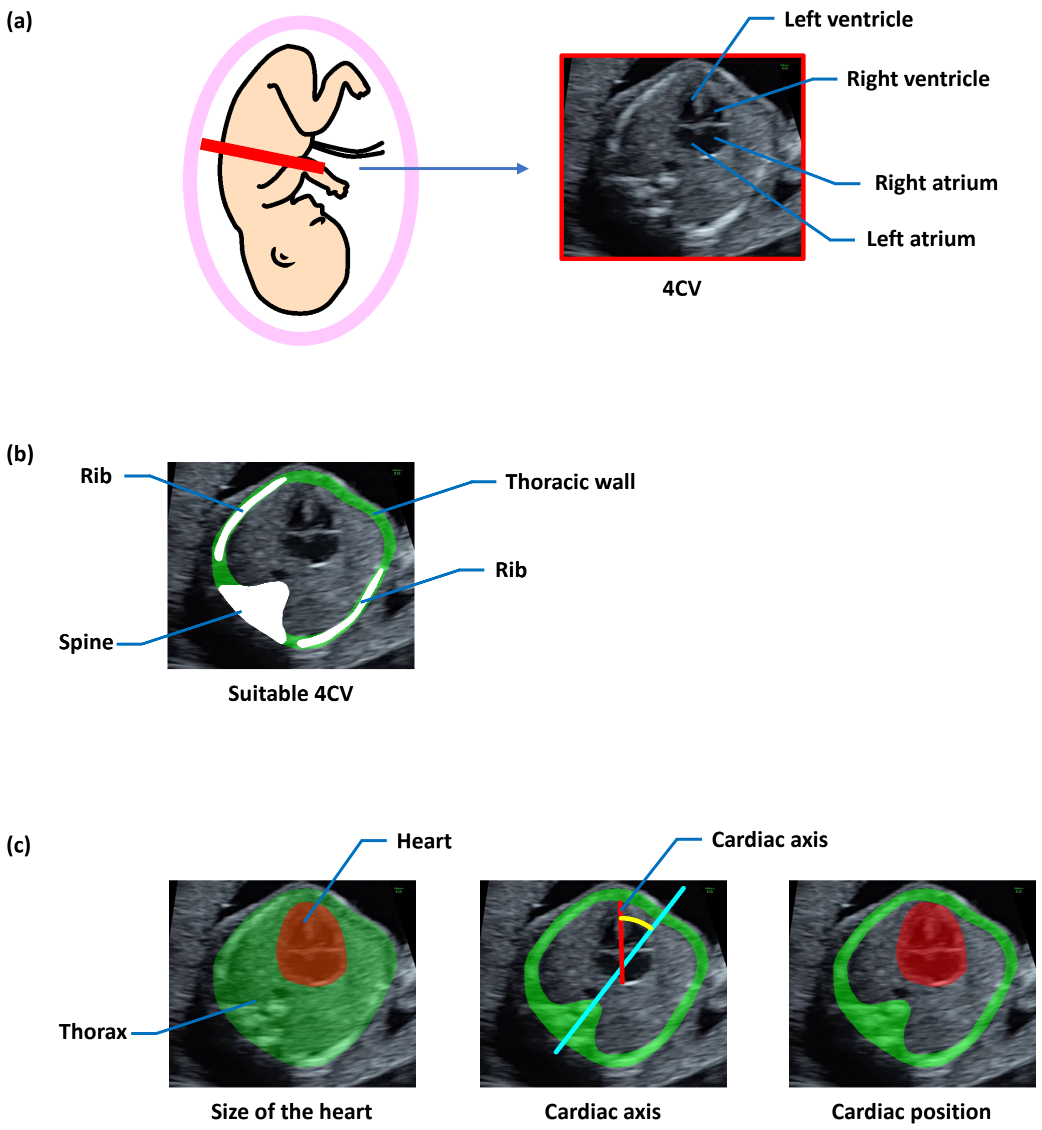

Supplementary Figure S1. Examples of clinical procedures that need to consider the thoracic wall when assessing 4CV in fetal ultrasound. (**a**) Schema of 4CV. 4CV is one of the cross-sections of a heart and contains four chambers—right atrium, right ventricle, left atrium, and left ventricle. (**b**) The examiners confirm that the obtained 4CV is suitable to assess by observing that the thoracic wall is close to circular shape and has a full length of a rib on each side. (**c**) Assessment indicators for 4CV examination. The size of a heart is usually less than one-third of the thorax area. Also, a normal heart is located on the left side of the thorax with its long axis pointing left by about 45 ± 20°. These indicators can trigger the detection of congenital abnormalities.

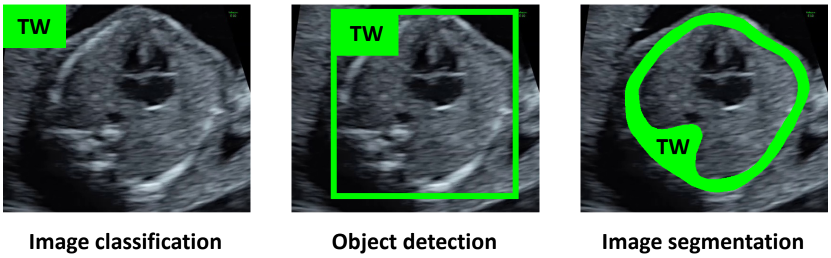

Supplementary Figure S2. The typical image processing methods using deep learning techniques. The figure shows the examples with TW as the object of image processing methods. Image classification classifies what the image is. Object detection identifies what is where in the image. On the other hand, image segmentation classifies what the individual pixels in the image are. Therefore, image segmentation provides detailed information about the object against the image, such as its orientation, size, and boundaries.

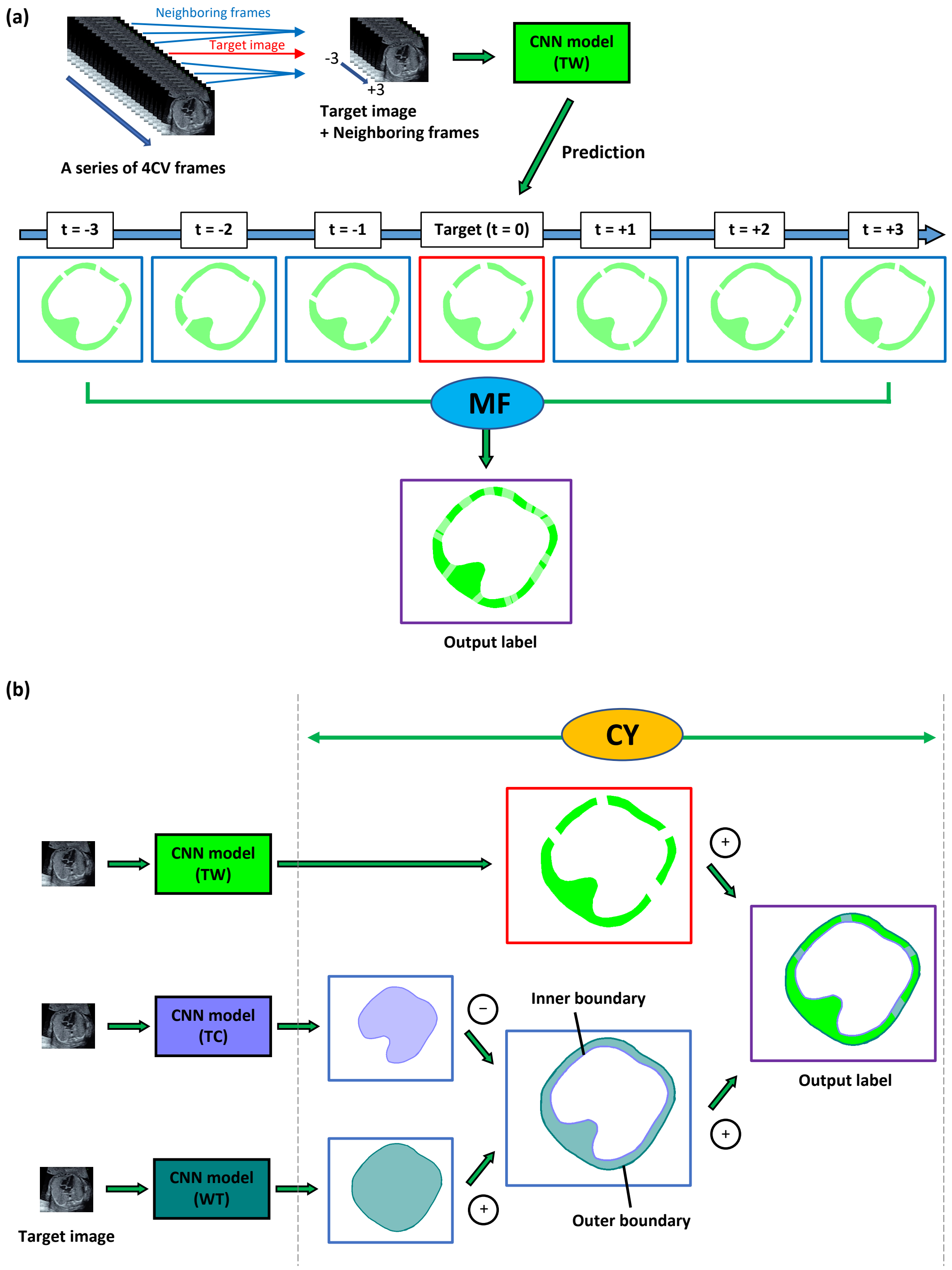

Supplementary Figure S3. Schematic diagrams of procedures for MF and CY. (**a**) The procedure for MF. The target image and its neighboring frames were extracted from a series of 4CV frames for MF. Then, the target image and its neighboring frames were transmitted into the trained CNN model to obtain the time-series multiple prediction labels of TW. MF integrated these prediction labels for a single TW output label. The time-series information could improve the segmentation performance. (**b**) The procedure for CY. Three prediction labels of TW, TC, and WT were prepared for CY. These three labels were obtained by transmitting the target image into each trained CNN model (TW, TC, and WT). CY integrated these three prediction labels into a single TW output label. In this integration, the prediction label of WT was subtracted by that of TC and added together to that of TW. CY could more firmly delineate the outer boundary and the inner boundary by this integration.

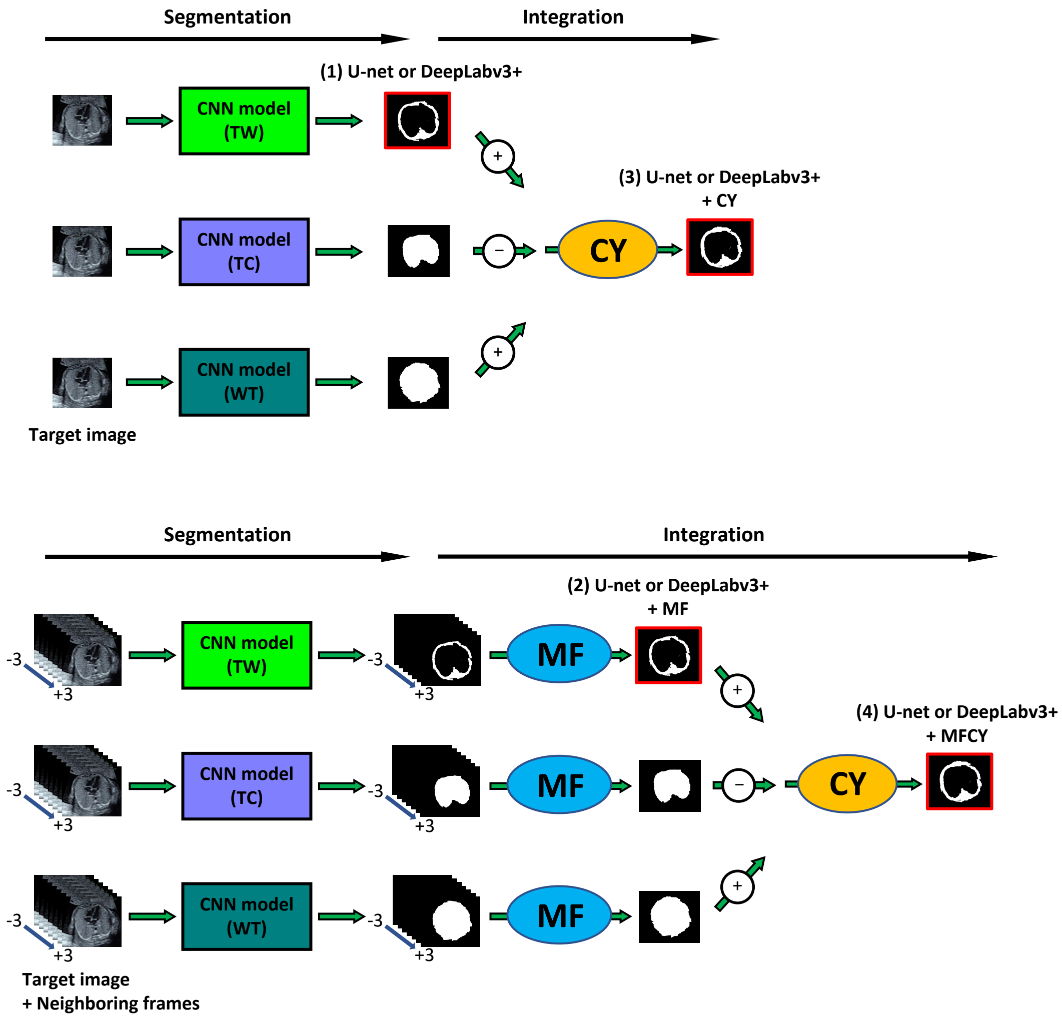

Supplementary Figure S4. Experimental diagrams for evaluation of MF, CY, and MFCY. We compared the segmentation performance of the following four methods: (1) U-net or DeepLabv3+, (2) U-net or DeepLabv3+ + MF, (3) U-net or DeepLabv3+ + CY, and (4) U-net or DeepLabv3+ + MFCY. (1) The prediction label of U-net or DeepLabv3+ was obtained from the simple use of U-net or DeepLabv3+. (2) The prediction label of MF was obtained from integrating the prediction labels of TW segmented from the target image and its neighboring frames. (3) The prediction label of CY was obtained by integrating three different prediction labels segmented from TW, TC, and WT. (4) The prediction label of MFCY was obtained by adapting the MF and CY, in that order.

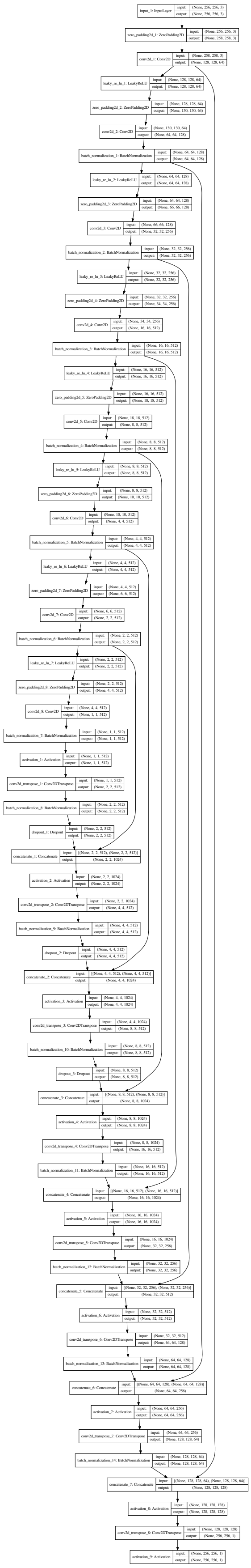

Supplementary Figure S5. Architecture of U-net.

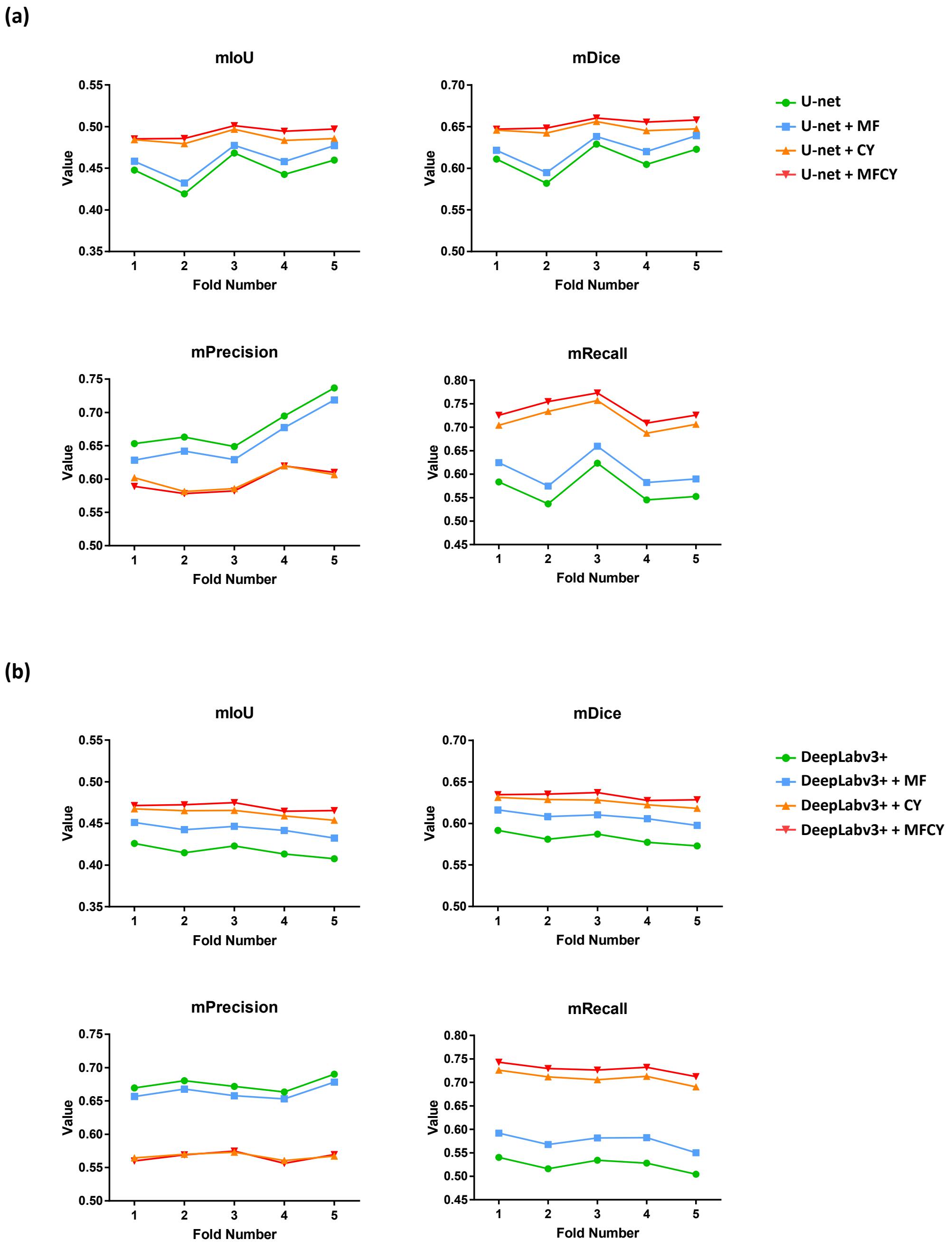

Supplementary Figure S6. Thoracic wall segmentation performance with five-fold cross-validation. The columns correspond to the values of mean intersection over union (mIoU), mean Dice coefficient (mDice), mean Precision (mPrecision), and mean Recall (mRecall). In each subfigure, the X-axis represents the number of folds and the Y-axis represents the value of the metric. The colored lines represent the segmentation method. CNN models were based on (**a**) U-net and (**b**) DeepLabv3+.

(a)

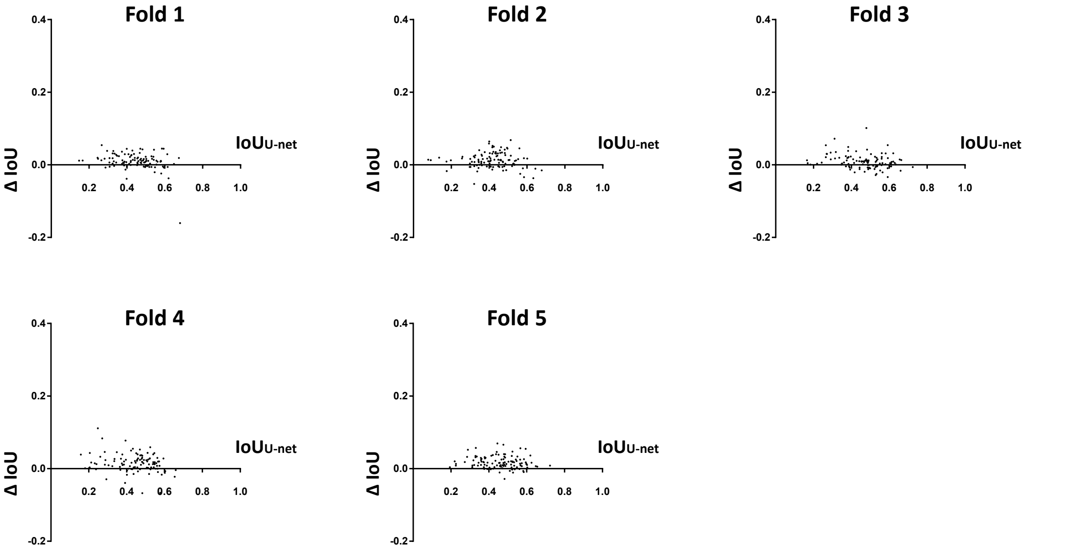

(b)

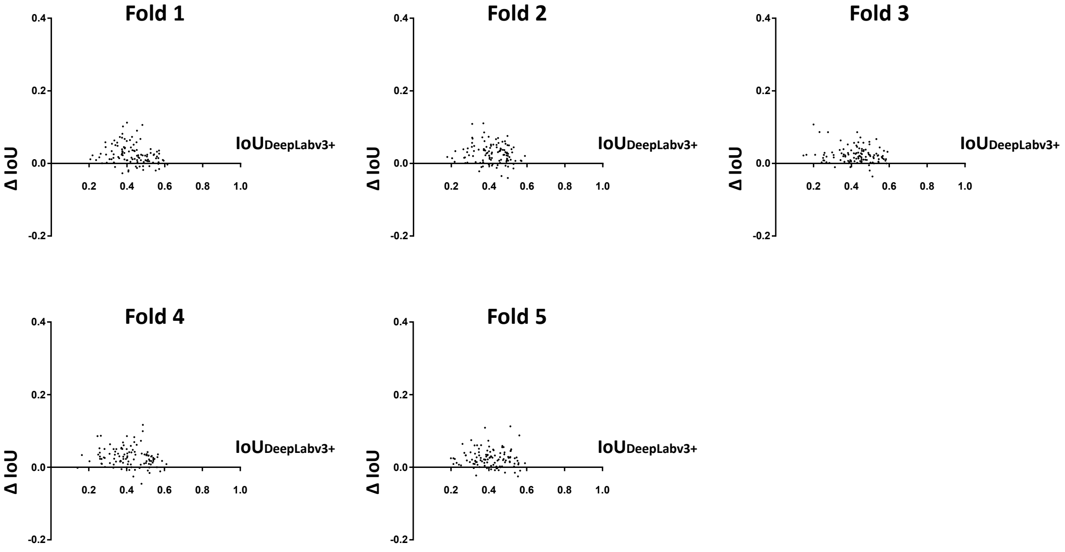

Supplementary Figure S7. The differences for the IoU values of the thoracic wall segmentation in individual test images in each five-fold using MF. Each subfigure shows each one-fold. The x-axis represents the IoU of thoracic wall segmentation of the single use of U-net or DeepLabv3+ ($IoU_{U\text{-}net}$ or $IoU_{DeepLabv3+}$), and the y-axis represents ΔIoU by MF. CNN models were based on (**a**) U-net and (**b**) DeepLabv3+.

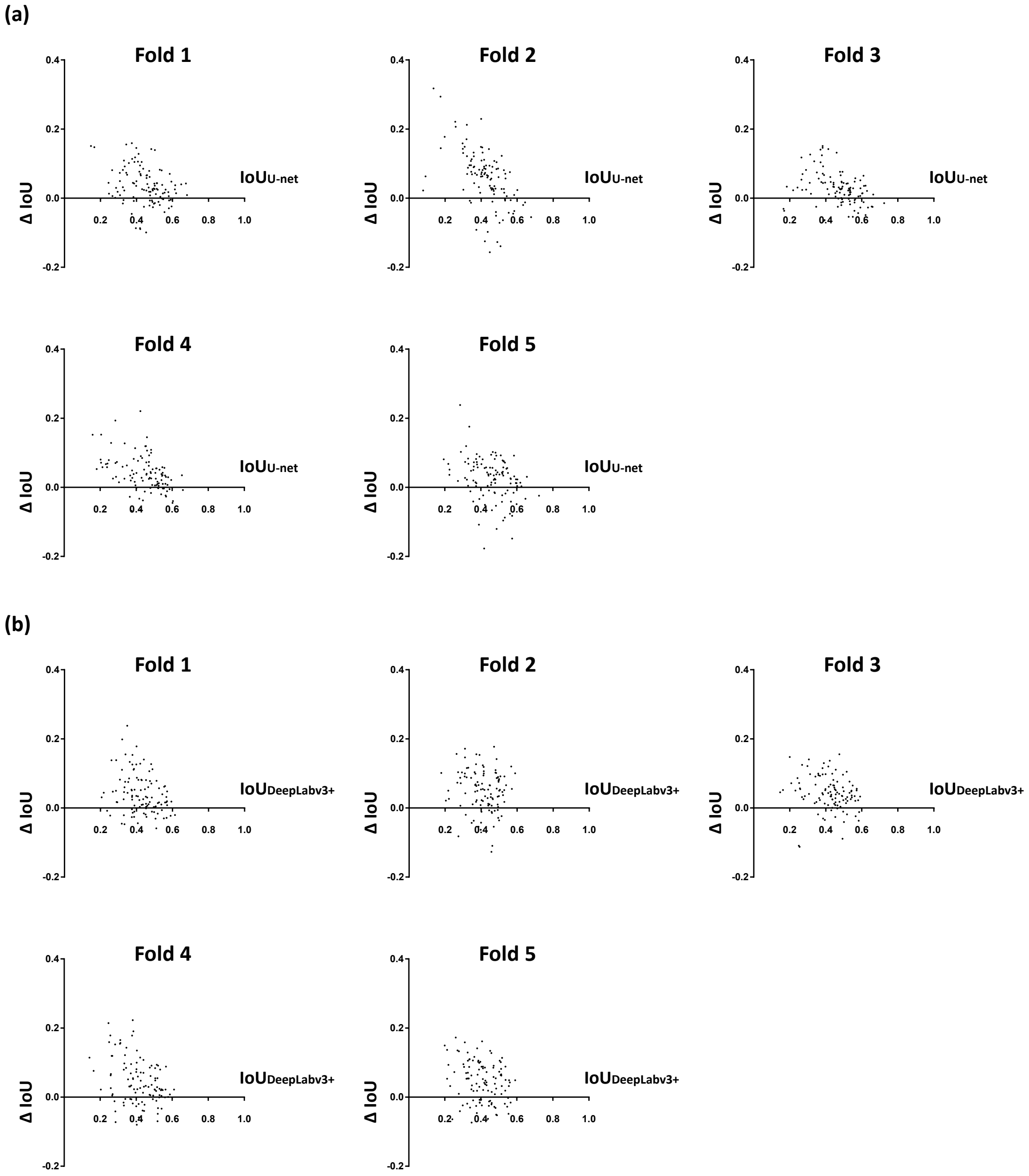

Supplementary Figure S8. The differences for the IoU values of the thoracic wall segmentation in individual test images in each five-fold using CY. Each subfigure shows each one-fold. The x-axis represents the IoU of thoracic wall segmentation of the single use of U-net or DeepLabv3+ (IoU$_{\text{U-net}}$ or IoU$_{\text{DeepLabv3+}}$), and the y-axis represents ΔIoU by CY. CNN models were based on (**a**) U-net and (**b**) DeepLabv3+.

Supplementary Table S1. The performances of MF corresponding to the segmentation of the thoracic wall, thoracic cavity, and whole thorax.

CNN	Structure	MF	mIoU		mDice		mPrecision		mRecall	
			Mean	SD	Mean	SD	Mean	SD	Mean	SD
U-net	Thoracic wall		0.448	0.017	0.610	0.016	0.679	0.033	0.568	0.032
		✓	0.461	0.017	0.623	0.016	0.659	0.035	0.606	0.032
	Thoracic cavity		0.856	0.004	0.922	0.003	0.943	0.012	0.905	0.008
		✓	0.864	0.006	0.926	0.004	0.936	0.013	0.920	0.007
	Whole thorax		0.843	0.029	0.912	0.019	0.954	0.005	0.880	0.036
		✓	0.852	0.022	0.918	0.014	0.950	0.005	0.894	0.028
DeepLabv3+	Thoracic wall		0.417	0.007	0.582	0.007	0.675	0.009	0.525	0.013
		✓	0.443	0.006	0.608	0.006	0.663	0.009	0.575	0.014
	Thoracic cavity		0.844	0.003	0.914	0.002	0.915	0.004	0.918	0.004
		✓	0.848	0.004	0.916	0.002	0.906	0.005	0.932	0.003
	Whole thorax		0.857	0.005	0.922	0.003	0.921	0.007	0.928	0.003
		✓	0.861	0.005	0.924	0.003	0.914	0.007	0.939	0.002

SD = standard deviation.

Supplementary Table S2. The performances of MF corresponding to the segmentation of the heart and lung.

CNN	Structure	MF	mIoU		mDice		mPrecision		mRecall	
			Mean	SD	Mean	SD	Mean	SD	Mean	SD
U-net	Heart		0.686	0.042	0.798	0.036	0.849	0.028	0.784	0.069
		✓	0.697	0.040	0.806	0.034	0.833	0.029	0.810	0.067
	Lung		0.712	0.015	0.828	0.011	0.832	0.020	0.832	0.014
		✓	0.713	0.015	0.829	0.011	0.808	0.021	0.859	0.012
DeepLabv3+	Heart		0.734	0.012	0.842	0.009	0.864	0.008	0.833	0.008
		✓	0.744	0.011	0.849	0.008	0.848	0.009	0.862	0.007
	Lung		0.684	0.010	0.808	0.008	0.833	0.008	0.793	0.014
		✓	0.697	0.008	0.818	0.006	0.814	0.009	0.830	0.011

SD = standard deviation.
